# Incident Traumatic Experiences and Poor Financial Wellbeing: A Double Hit for Cognitive Aging?

**DOI:** 10.1093/geroni/igaf122.937

**Published:** 2025-12-31

**Authors:** Katrina Kezios, Eleanor Hayes-Larson

**Affiliations:** Boston University, Boston, Massachusetts, United States; University of Southern California, Los Angeles, California, United States

## Abstract

Traumatic experiences and financial hardship may jointly affect cognitive aging through shared stress-related pathways. Using data from the Health and Retirement Study (N = 3432), we quantified the effect of incident traumatic experiences (ITE, occurring 2006-2010 or 2008-2012), defined as experiencing ≥1 events from the Lifetime Traumas index (death of a child, major natural disaster, firing/being fired upon in combat, spouse/partner/child addicted to drugs/alcohol, serious physical attack/assault, life-threatening illness/accident, or life-threatening illness/accident in spouse/child). We examined effect modification by poor financial wellbeing (FWB), defined as reporting ≥3 of 7 subjective (financial dissatisfaction, low financial control, difficulty paying bills, taking less medication because of money) and objective (low income, low non-housing wealth, no housing wealth) indicators of financial hardship. Memory was assessed biennially from 2010/2012 to 2020 via a composite score computed from direct memory assessments and proxy reports. We used linear mixed models to estimate the effect of ITE on ∼10-year memory decline overall and stratified by FWB. Models used follow-up time as the timescale and adjusted for baseline age, practice effects, sex/gender, race, educational attainment, parental education, Southern birth, childhood SES, marital status, self-rated health, and interactions between covariates and time. Overall, having ≥1 ITE was associated with accelerated memory decline (β_anyITE*time=-0.007, [95% CI:-0.012, -0.002]). The magnitude of this association was larger in the presence of poor FWB (β_anyITE*time|FWB≥3=-0.014, [-0.030, 0.003] vs. β_anyITE*time|FWB < 3=-0.006, [-0.012, -0.001]). Our results suggest financial wellbeing may modify the effects of traumatic experiences and may be a policy intervention point to delay memory decline in older adults.

